# Consent and assent in paediatric research in low-income settings

**DOI:** 10.1186/1472-6939-15-22

**Published:** 2014-03-05

**Authors:** Phaik Yeong Cheah, Michael Parker

**Affiliations:** 1 Mahidol Oxford Tropical Medicine Research Unit, Faculty of Tropical Medicine, Mahidol University, Bangkok 10400, Thailand; 2 Centre for Tropical Medicine, Nuffield Department of Clinical Medicine, University of Oxford, Oxford OX3 7LF, UK; 3 The Ethox Centre, Nuffield Department of Population Health, University of Oxford, Oxford OX3 7LF, UK

**Keywords:** Consent, Assent, Paediatric research, Low-income setting, Children

## Abstract

**Background:**

In order to involve children in the decision-making process about participation in medical research it is widely recommended that the child’s assent be sought in addition to parental consent. However, the concept of assent is fraught with difficulties, resulting in confusion among researchers and ethics committees alike.

**Discussion:**

In this paper, we outline the current international debate surrounding pediatric consent and assent, and its unique challenges arising in low-income settings. We go on to propose some key requirements for a fit-for-purpose assent model in these difficult settings. The paper recommends that children who are competent, that is, children who are judged to be able to understand and retain relevant information, weigh this information in making a mature judgment, come to a decision and communicate the decision, should be able to consent for themselves. Our proposal is that where the decision about whether to participate in a study is of comparable complexity to the decisions the child is used to making in other aspects of his or her life, it should be made by the child him or herself. The relevant level of complexity should be judged by local standards rather than standards of the developed world. In the paper we explore some of the practical challenges and counter arguments of implementing this proposal. As in high-income settings, we argue that in the case of children who are judged to lack this level of competence both parental consent and assent from the child should be sought and go on to define assent as *involving* the child to the extent compatible to his or her maturity and with cultural norms and *not* as obtaining the child’s permission to proceed.

**Summary:**

The concept of assent in the current guidelines is confusing. There is an urgent need for clearer guidelines that can be adapted for all types of paediatric research wherever it is to be carried out and an evidence-base concerning good assent/consent practice. This paper argues that a context specific approach should be adopted when assessing whether consent or assent should be sought from children in low-income settings.

## Background

### Research in children

Conducting medical research involving children is important and necessary. It has the potential to lead to innovations in healthcare that can substantially improve their health and quality of life. The physiological differences between children and adults mean that often it is not sufficient, scientific or ethical to carry out research with adults and apply the findings to children. What this means is that there are good reasons for high quality, ethically robust, medical research in children to be supported and encouraged. Such research is likely to need to include systematic investigation into childhood development, and the aetiology of childhood diseases, as well as careful scrutiny of the means of promoting health and of diagnosing, assessing and treating disease in children. In many cases, it is also important to validate in children the beneficial results of research conducted in adults and making dose adjustments for medications proven effective for adults.

Conducting trials that meet international standards in low-income^a^ settings where there is often a lack of basic infrastructure, difficulty accessing adequate or even basic healthcare, low levels of education, high levels of illiteracy, poverty and high prevalence of diseases of poverty like infectious and parasitic diseases, and nutritional deficiencies is challenging. Research with children in such settings is, however, desperately needed. Nearly 7 million children under five years old died in 2011; nearly 800 children every hour [1]. There is clearly a need for more research that directly benefits children in low-income settings. However, whilst children and young people make up a significant proportion of the population in the developing world, they are under-represented in all areas of clinical research.

Whilst medical research in children is important for these and other reasons, children require special protection because they may be less likely than adults to be able to express their needs or defend their interests. Whilst it is important not to over-generalise, children who are ill can be frightened, overwhelmed and distressed by their illness and by healthcare contexts, and this may be compounded by the introduction of additional research-related procedures and visits to clinics. Childhood can be a vulnerable, formative time, when harms can have serious impact as well as being potentially long lasting.

There has been much discussion in the literature and in the guidelines about the ethical issues arising in research with children [2]. The issues identified and discussed include: levels of acceptable risk; the balance of risks and benefits; confidentiality; the role of parents and people with parental responsibility; and the relationship between therapeutic and non-therapeutic research. Perhaps the most enduring and widely discussed aspect of research in children has concerned issues related to consent and, in particular, how to develop an appropriate model of decision-making which takes into account the importance of involving research participants in decision-making, the developing autonomy and maturity of children and young people, the special obligations and rights of parents, and the additional obligations of health professionals and researchers. In this context, one particular concern has been how, to what extent and in what regard decision-making about research participation of children who lack full decision-making capacity should require the involvement of these children. Increasingly, views about best practice in such cases, and paediatric research guidelines, have emphasize the importance of what has come to be known as “assent”.

Whilst there has been a significant amount of discussion about assent in high-income settings and some discussion about how this might apply in low-income settings, there has been very little exploration of the ways in which assent arises as a practical ethical challenge in the design, conduct and review of research in such settings. In this paper we draw upon our own experience of establishing and managing research with children (PYC) and providing ethics support in paediatric research (MP) in Africa, South East and South Asia to begin to map out some of the ways in which consent and assent have arisen as practical problems. These include but not limited to large multi-centre clinical trials on severe malaria [3], uncomplicated malaria [4], melioidosis and other tropical diseases (http://www.tropmedres.ac). Our trials have been and are largely conducted in rural communities with low-income levels, both in indigenous and non-indigenous populations, stable and mobile communities, lasting one to three years, and usually involve marketed and generic drugs. These trials are of minimal to moderate harm as they do not involve early phase drugs or any experimental procedures.

A typical example to illustrate the consent and assent problem is a study we are currently conducting in Bangladesh in which children who had severe malaria as babies and toddlers are followed up and assessed using psychometric tests for the presence of long-term neurological impairments [trial registration: ISRCTN73295852]. These children are now in their teens. As a part of the local requirements we have to seek assent from these children in addition to their parent’s consent. Field workers have found it uncomfortable to seek assent from these them once their parents have consented, as it is deemed culturally inappropriate to go against their parents’ wishes. On the other hand, they feel that older children should be treated like adults and should be able to consent for themselves. Some of the female children are already married and have their own children although they are below 18 years old. These issues are not uncommon. They occur on a daily basis in most of our studies, at most of our sites.

Clearly there is a great deal of diversity both in and between low-income settings and this paper is based on a limited range of studies. However, whilst acknowledging this diversity and recognizing that work needs to be done on these issues our purpose in this paper is to outline and explore some of the most important differences between high and low-income settings as they have arisen in our own work.

## Discussion

### Assent as a proposed solution to involving children in research ethically

Most established paediatric research guidelines require that assent should be sought from children for participation in research in addition to the seeking of their parents’ consent. For example, the UK Royal College of Paediatrics and Child Health guidelines state that, “When parental consent is obtained, the agreement of school age children who take part in research should also be requested by researchers” [2]. The US Code of Federal Regulations states that, “the IRB must determine that adequate provisions are made for soliciting the assent of the children when in the judgement of the IRB the children are capable of providing assent” [5]. Many middle and low-income countries also advocate that assent be sought from minors, for example Bangladesh (not available online), South Africa [6], Thailand [7] and Malaysia [8].

Arguments for the use of assent in the recruitment into research of children who do not have the capacity to consent tend to call upon two distinct and important reasons – instrumental and moral. For instrumental reasons, it is argued, assent is important because it is a way of helping children to feel comfortable with participation and hence stands a better chance of ensuring that children take medicines as required for the study, attend clinic visits, report any adverse events and so on. It also offers an opportunity for the identification and addressing of the child’s concerns: concerns of which the adult providing consent may not be aware. For example, they may not want to miss school to attend follow up visits for their own reasons or they may be scared of injections. In addition to these instrumental reasons, but related to them in important ways, is the role assent can play in treating children with appropriate respect as a person with interests, values, concerns and so on. It is, that is to say, a way for researchers and health professionals to acknowledge and respect his or her emerging autonomy.

### Problems with assent in medical research

While there is a broad consensus that assent should form part of the process by which children are involved in paediatric clinical research, there continues to be much uncertainty about its precise meaning and how it should be integrated into research practice. This results in a significant amount of variability in practice and confusion among researchers and research ethics committees about what constitutes good practice. There are a number of reasons for this.

To some extent, this arises from the fact that the meaning of assent is not clearly articulated in the guidelines. For example, whilst the US Code of Federal Regulations states, “assent means a child’s affirmative agreement to participate in research” [5], other guidelines take assent to mean variously: “acquiescence” [2,9], “knowing agreement” [10] or “active agreement’ [11]. In addition to their variety, the conceptualizations of assent are also problematic because they fail to make a clear distinction between assent and consent: if a child is deemed incompetent and unable to consent to participate in a study, how is it that he or she can be judged able to provide “affirmative agreement”? Whilst the meaning and the requirements for *consent* for research are reasonably clear, what is meant by agreement or acquiescence in the context of assent is not defined in ways which can be clearly distinguished from consent.

Secondly, important difficulties arise in relation to how assent should be obtained in practice. International guidelines suggest that the child should be informed about the trial to an extent compatible with his or her understanding and, if capable, he or she should sign and personally date the informed assent form or assent certificate [12,13]. They recommend that researchers use children information sheets and assent forms that are in a similar format to typical adult information sheets and consent forms. But this is a requirement that can cause practical problems. These written documents test the child’s linguistic skills and reading abilities rather than understanding or comprehensibility. More importantly, this requirement causes the misunderstanding that assent has the same significance as consent and further compounds the lack of conceptual clarity highlighted above.

Thirdly, although the current assent guidelines are right in our view to avoid setting agreed lower and upper age limits in the assent process (and lower age of consent), this also inevitably creates a degree of uncertainty in practice. Most guidelines suggest that children should assent to enroll in a study where appropriate. The UK’s Royal College of Paediatrics and Child Health guidelines, suggest that investigators should consider seeking assent from school going children [2]. Other guidelines suggest assent should be sought from age seven and older [11,14]. While these guidelines give researchers flexibility, the fact that they may be interpreted in a number of ways can leave researchers and research actors such as fieldworkers, uncertain about what the right thing to do is.

Fourthly, the recommendations stress that dissent especially *sustained* dissent should be respected [15]. This is problematic because if dissent can overrule parental consent then the parent is in fact not providing consent. Conversely, if a child wishes to participate in a study and the parent does not want them to, then assent has no decision-making power. The situation is further complicated by the fact that the guidelines do not define what dissent means as there is clearly a difference between a child not wanting to participate because he or she simply does not want an extra injection, and refusing because the he or she is too young to understand the purpose of the study or altruism.

Taken together, this means that the current guidelines on assent inevitably have the potential to generate a considerable degree of uncertainty and disagreement about what constitutes best assent or consent practice. This presents a number of difficulties in practice for both researchers and for research ethics committees regarding how such guidelines and principles should be interpreted in particular cases and settings. These challenges are particularly difficult in the context of research in low-income settings where decisions about the inclusion of children in research presents problems not encountered in combination elsewhere.

## Points to consider when assessing whether consent or assent should be required from a child for research participation in low-income settings

Assent is also advocated in the guidelines relating to research with children carried out in low- income settings. The Declaration of Helsinki, for example, states that where a potential research subject deemed to lack the competence to give valid consent is capable of assent, the physician must seek that assent in addition to the consent of the legally authorized representative. The Declaration also states that the potential subject’s dissent should be respected [16]. The World Health Organisation Ethics Review Committee (WHO ERC) guidelines state that “while the age at which this informed assent should be taken varies, researchers should consider asking for assent from children over the age of seven years with assent taken from all children over the age of twelve years” [14].

Inevitably, along with the other challenges presented by research with children in such contexts, the problems of what constitutes good assent and consent practice are magnified in low-income settings and researchers and ethics committees struggle to implement the requirements of international guidelines. This means that inevitably in some cases such requirements are applied over-rigidly and in others they are not applied at all. This suggests the need for the development of models of good assent and consent practice applicable in the context of the challenges presented by low-income settings.

A key step in this process is the identification of the nature of the challenges presented. In what follows, based on our experience conducting trials in low-income settings, we outline nine key factors that will need to be taken into account in any adequate approach to assent in research with children in these settings. We then go on to outline some of the elements of an approach to decision-making practice capable of taking these factors into account.

### Lack of formal education and high illiteracy rate and/or earlier maturity

Children who live in poverty may not have the same level of formal education and exposure to concepts relating to science and medicine as children in high-income settings. Many parents and their children are illiterate and may not have basic knowledge of how the body works or how disease occurs. On the other hand, some children may grow up faster in poverty, may be more mature and street-wise and may be taking significant decision-making responsibility in their everyday lives. They may, for example, be looking after younger siblings or helping support the family by way of earning money or helping with family work be it at home or in the farm. At a young age, they may be independent and used to taking on more responsibilities and making important decisions in their lives than children in higher income settings. Some children are married and are themselves parents or the sole carers of siblings or other children. The special status and experience of these “emancipated” minors is recognized in some countries although not in others.

### Children may be better informed than their parents

In some low-income settings, children may have more opportunities for education and be more exposed to technology than their parents. As a consequence, it is not uncommon for children in these settings to be more educated than their parents. These children can read and write, understand medicine and health better than their parents because they have had the opportunity to go to school, are more connected to the wider world and have access to information via the web, telephones and other means.

### Lack of familiarity with medical research

In low-income settings, many people will be unfamiliar with medical research or with the guidelines that govern such research and few would have had personal experience of consenting to a research study. Furthermore, the difficulty of distinguishing between medical research and routine clinical care in such settings has also been well-documented [17]. Against this backdrop, the concepts of consent and assent are likely to be both unfamiliar and difficult to grasp, particularly in situations where many patients will be used to leaving decisions about their medical care to their doctor, and unused to making medical-related decisions. Obtaining assent in addition to consent is likely to cause confusion not only among patients but also the local research team. A researcher asking for assent may be viewed with suspicion and this may adversely affect the patient-doctor relationship.

### Hierarchical societies

Often children in more conservative hierarchical societies are not taught to speak up as they “should be seen and not heard”. This is different from societies where even very young children may be nurtured to develop their own views. In traditional families, there is more parental control, the child’s rights and interests may be socially undervalued or seen as inseparable from those of adults. Furthermore, because a decision to take part in a research study often affects the whole family, for example in relation to the family economy, the decision might ultimately be made by the head of the family who is often the elder male or the breadwinner of the family, not necessarily the child’s biological parent. In such contexts, seeking child assent might be seen by community members as absurd at best and insulting at worse.

### Complex family relationships

Complex family relationships in traditional families can pose problems for the seeking of parental consent and childhood assent. It is not always clear, and certainly rarely put in writing, who has formal responsibility for a child – it is not necessarily the biological parent. The concept of “legally authorized representative” as stated in some international guidelines for those without biological parents rarely makes sense in most low-income settings [16]. A household of twenty people may have six adults and fourteen children, and all six have somewhat equal responsibility over the fourteen children regardless of their blood relations. The term “aunt” or “uncle” is loosely used. One should question who is eligible to give consent for these children – who has the responsibility for the child’s best interest. This is particularly acute in settings where great distances, lack of communication infrastructure and social dislocation may serve to make biological parents unreachable. One example is our field research site on the Thai-Burmese border, which is an area of convergence of largely ethnic Karen refugees and migrant workers who cross into Thailand for employment and other activities (http://www.shoklo-unit.com). The population is highly mobile in that it moves between the two countries and some have been resettled to third countries. One of the challenges of conducting paediatric research there is addressing the complex issue of consent and assent for example, who can legitimately provide parental consent and who has the child’s best interests.

### Problems with documentation

In research, the view is often taken that what has not been documented did not happen. As a consequence, in order to prove that assent has been obtained, most guidelines require that an “assent form” be signed and dated by the person taking assent as well as by the child, and if the child is illiterate, a witness (not the parent) should sign on the child’s behalf. Although this may seem sensible in the developed world, it might be viewed with suspicion and as overly bureaucratic in traditional communities where signing is something not usually done even by adults. Some communities are suspicious of signing a document because they had “lost land because of signing a document” [18].

Many hospitals in low-income settings do not keep good medical records. In most guidelines it is recommended that age-appropriate children’s information sheets are created for children. For example, in the UK, one can end up with up with a requirement for four sets of information sheets and consent/assent forms for one simple study i.e. adult information sheet, parent information sheet, information sheet for children aged 7 to 12 years and information sheet for children aged 13 to 17 [13]. This is made worse in less informed populations where it is even difficult to provide written adult information sheet translated to local lay language. This has the potential to be either (or both) an unrealistic expectation or an administrative burden out of proportion with the practices in the settings. The researchers’ need of ensuring that there is proof of assent and their effort to allay the fears of the parent and child who have to sign on a piece of paper may distract the researchers from more important ethical and medical issues.

### Studies in low-income settings are different

The types of paediatric research conducted in low-income settings are often different from those conducted in high-income settings. In high-income settings, studies tend to be smaller, early phase product registration studies and studies in non-communicable diseases conducted in well-equipped hospitals. In low-income settings, paediatric research is often larger and tends to be more disease management type research. If such research involves drugs, it tends to focus on the use of registered products or generics conducted mainly to help relieve the burden of disease in that community [19]. It tends, furthermore, to be conducted in resource-starved hospitals with lower doctor to patient ratios and is, hence, more likely to be the case that enrolling in the study is in the best interest of the child - for access to drugs, diagnostic tests or medical expertise.

### Urgency and emergency situations

Like adults, children in low-income settings may have acute medical conditions, such as severe malaria which affects millions of poor children every year, requiring immediate treatment. The International Conference for Harmonisation Good Clinical Practice (ICH GCP) guidelines make allowance for a waiver of consent provided that consent can be provided by the subject’s “legally accepted representative” [20]. However, in low-income settings, it is very rare for a child, or an adult, have a “legally accepted representative”. In such situations, ICH GCP requires following of a protocol agreed with the relevant ethics committee and that the legally accepted representative should be notified as soon as reasonably possible. For research conducted in emergency or desperate situations, seeking assent from a child may be viewed by such bodies and by local researchers and health professionals as a hindrance, time consuming and potentially harmful.

### Poor regulations

Regulations for research in children in developing countries are often rigid, confusing or non- existent. Although research ethics committees exist in most countries, they rarely offer clear guidelines for research in children. Those which do tend to adopt international guidelines, with the noble intention of protecting children but with little reflection on their relevance to the local setting, resulting in practical problems for the conduct of the research. For example some countries adopt the ICH guidelines which recommend written assent for children, without contexualizing their implementation. These problems are magnified in studies that are multi-sited, conducted across different cultural and economic settings. In some of these cases, assent is obtained for the wrong reason – merely to ensure that the regulatory box is checked.

## The way forward – a framework for consent and assent in research with children in low-income settings

It is clear from the discussion above that there is a pressing need for the achievement of clarity and consistency in the guidelines and for further empirical research to be carried out to inform the development of an evidence-based approach to best practice in the implementation of models of assent in particular settings. The globalization of paediatric research suggests that this work and any effective framework will need to be both based on sound ethical guidelines and also to be capable of taking into account the diverse contexts in which such research takes place including local cultural, medical and legal factors. Current guidelines have significant limitations which often leave researchers and ethics committees uncertain about best practice and consequently mean either that important research is not being carried out or in some cases that research may be proceeding in ways which might come to be seen in retrospect as unethical [21].

Bearing in mind the considerations above, it is our view that an adequate framework for thinking about consent and assent will be one be informed by the following *prima facie* principles: that children who are competent (where competence is understood as relative to a specific decision in a specific context and judged against the background of locally accepted decision-making practices and expectations) should consent for themselves; and, that in the case of children lacking decision-making capacity (similarly contextualized), both the child’s assent and parental (or locally relevant alternative) consent should be obtained. In our view, the key moral difference in decision-making about research participation is competence - where in the absence of a gold standard for competence assessment in children, competence might perhaps be provisionally defined as including the ability to understand and retain the relevant information provided, the ability to use this information make a decision, and the ability to communicate the decision.^b^ Competence is task- and context-specific, which means that assessment of competence should be regarded as a specific judgement at a specific moment of the ability of the individual to fulfil a task that he is facing [22].

These principles and some of their limitations and counter arguments are discussed further below.

### Competent children should be able to consent to or refuse participation for themselves

Whilst relating to chronological age and physical and mental development, the concept of childhood also relates to varying and contextually framed ideas about responsibility, vulnerability and competence, which can vary between cultures, nationalities, and according to socio-economic status [23]. Notwithstanding the existence of a degree of variability, the age of eighteen is widely legally accepted internationally as marking the start of adulthood and as the age at which children assume legal responsibility, can vote, get married and so on [24]. However in some countries, where guidelines exist, the legal age to work, drive, buy alcohol, have sexual relations and seek medical care can be younger. Furthermore, in practice, the responsibilities, social roles and decision-making expectations allocated to children can be significant. The adoption of fine-grained age-related distinctions is inevitably to a degree arbitrary: there is no reason to believe that the cognitive ability and maturity to competently make independent decisions correlates with a particular age. Whilst recognizing that there are limits to this, it is nonetheless clear that in many cases, children much younger than, say, eighteen have the capacity to make important decisions, including those relating to health care, of a similar level of complexity to adults [25]. Whilst acknowledging the pragmatic importance of adopting policies, which can be implemented, it is vital that such policies and the approaches to decision-making they require are reasonable and justifiable in relation to those who are going to be affected by the resulting decisions. What this suggests is that when considering research with children in low-income settings, researchers and ethics committees should review the cut-off age of consent for a given study to ensure that it is culturally and context specific and bears a reasonable relation to the likely competence of children to be recruited, in addition to being effectively action-guiding and usable in practical recruitment settings.

The default position should be that those who are competent should be able to consent (or refuse) to participate for themselves regardless of their age. A competent child who can make his or her own autonomous decisions should be allowed to do so – the required level of competence being relative to a specific decision [26]. The decision about whether to participate in a study, if it is of comparable complexity to the decisions the child is used to making, should be made by the child. This complexity should not be judged according to first world standards, but in relation to the local setting taking into consideration the benefits, risks and implications of the available choices the child can usually or sometimes make.

### Assent should be sought from incompetent children in addition to the obtaining of parental consent

In cases involving the recruitment of children *without* decision-making capacity, parents or “persons with parental responsibility” – which is a status that needs to be developed and agreed in the light of local practices, norms and expectations - should consent to (or refuse) participation on behalf of the child, such decision being made in the child’s best interest. Any such decision should take into account, to the extent these are discernable: the child’s wishes, fears, desire for altruism and likelihood of compliance to procedures in addition to the usual considerations when assessing best interest. If possible, the researcher should encourage both parents to take part in the decision making process as it has been shown to increase the extent to which consent is truly informed by the range of considerations relevant to the child’s best interest [27]. There will be some situations where parents will not act in the child’s best interest e.g. refusing because of other family commitments when enrollment in the study is the only way to access drugs, diagnostic tests or medical expertise; or conversely enrolling the child because the compensation offered is attractive for the parent or family. In such situations, the child’s best interest should prevail [24]. Parental consent (or refusal to consent) will probably not be valid if it is given against the child’s interests [2]. For children without decision making capacity, the parent should provide written informed consent by way of signing and dating the parent consent form the usual way.

In the recruitment of children lacking decision-making capacity into research the child’s assent should also be sought. It is our view that, contrary to established international guidelines, assent should not be conceptualized as seeking the child’s agreement or permission. We are in agreement with those who argue that assent should be understood as the range of practices through which the researcher engages with and involves the child, encourages the child to ask questions, treats the child with respect and acknowledges and supports the development of his or her emerging autonomy [28-30]. It is not consistent with the assessment of the child as “lacking competence” for the child to be asked to make the decision or “agree” or “acquiesce” as advocated in current guidelines. In such situation the decision should be made by the relevant decision-maker but complemented by the seeking of assent from the child, where assent is understood as a process of respectful and sensitive engagement and involvement. Assent as we define it should rarely be waived in the context of research and should not have any lower age limit. The communication strategy should be context specific and appropriate to the child’s needs – it is likely to be verbal or to involve the use of pictures, diagrams, videos or alternative media rather than a formal written document with a title and paragraphs as is sometimes currently the case. The extent of any involvement inevitably and appropriately depends on the maturity of the child, the relationship between the parent and the child and the norms of the culture.

## Judgement and accountability

In practice, for any given study and given site, the research team and ethics committees, in consultation with other relevant stakeholders such as perhaps community leaders or community advisory boards, will need to come to an informed view about the appropriate approach to consent and assent in any particular project, and safeguards from abuse should be in place. In many cases, for pragmatic reasons, we recognize it may be appropriate for the chronological age be used as a proxy for capacity. However, any such decision will need to be documented and justified and scope will need to be left for those who recruit such children to make judgements in particular cases e.g. where younger children have capacity or those who are older do not. Whichever approach is adopted in a particular case in relation to consent and assent and the relationships between them, such policies and judgements should be clearly justified, recorded and approved by relevant parties. This is likely to be led by local ethics committees which is in line with some of the existing guidelines for paediatric research regarding assent e.g. the US Code of Federal Regulations (Part 50) state that “the IRB must determine that adequate provisions are made for soliciting the assent of the children” [5]. The local ethics committees in consultation with other local stakeholders should have a policy of the minimum age of consent and the types of studies that children younger than the age of majority can consent for, and the appropriate way of documenting that assent has been sought for example through audio or video tape as opposed to signatures [31]. Such decisions should be reasonable and transparent and subject to scrutiny.

The assent process should be documented by the researcher not the child i.e. the child should not have to sign on a form similar to a consent form but the researcher should document it in the medical notes or case report forms that assent has been sought and a copy of the documentation should be filed in the investigator file. A good justification should be provided for why a waiver of the signature of the child participants is needed and this justification should be clearly written in the protocol and approved by the relevant ethics committees. It is our view that the obtaining of written informed assent where both the researcher and the child sign, date and print their names, as recommended by most guidelines, is inappropriate.

## Some possible counter arguments

Whilst the varied and complicated contextual aspects of carrying out research in low-income settings will inevitably present important challenges to any approach to the recruitment of children and young people in research, it is our view that the principled approach outlined above offers the basis for a coherent and culturally appropriate approach to such recruitment. There are, however, a number of possible objections to the position outlined. Some of these possible objections and our responses to them are as follows.

1. There is increased potential for tensions to arise between parental consent and the child’s refusal.

As in all medical care involving children, negotiations between the parent and child are unavoidable and part of good medical practice. The approach outlined above is sensitive to this aspect of medical practice. If a child who lacks the capacity to consent dissents from participation, this should be taken seriously and most current guidelines support this position. In the case of a competent child (or emancipated child), it is our view that he or she should able to refuse to participate irrespective of the parent’s wishes. Clearly it would be good practice to encourage discussion and to facilitate agreement where possible. Ultimately however, we believe the final decision should rest with the competent child or young person.

2. The proposed approach goes against local norms and guidelines.

Sensitivity to cultural norms is an important part of ethical research practice. However, there are limits to this. It is our view that those who are competent, judged according to the criteria outlined above and the development of policies subject to local ethics committee approval, should be able to make their own decisions about whether to participate or not to participate in medical research. The age of person is irrelevant to this. Seeking “agreement” from incompetent children once their parents have consented, as currently advocated is equally culturally inappropriate in some settings for example in rural Bangladesh as illustrated previously. It is our view that empirical work needs to be done in order to feed into the current existing international and country specific guidelines

3. The proposed approach may be seen as relaxing the requirements for protecting young research subjects.

It is our view that the opposite is the case. The interests of the individual competent research participant are enhanced, in research that meets international standards e.g. where there is no greater than minimal risk, where valid consent is obtained from the research participant himself or herself. In the current model, consent is obtained from parents even where young persons are fully competent. It is our view that the current model has the potential to fail to adequately protect the interests of young people. Given the complexity of family life outlined above, to take one example, there can be no guarantee that the consent of the biological parents adequately captures the interest of the competent young person.

4. It might be difficult to ensure in practice that researchers are not exaggerating the competence of young people in order to avoid the obligation to obtain consent from parents.

Given the pressures on frontline research staff to meet recruitment targets and the commitment of research institutions to successful research, it might be argued that there may be pressure on those who recruit young people to research to exaggerate their competence in order to save time and to avoid the need to seek additional consent from a legally recognized adult. This risk is important. Were it to lead to children lacking competence being recruited to research without adequate protections this would be an important worry. In our view, it is equally morally worrisome not to seek consent from a competent young person. In any system of consent for research there is always the potential for abuse and there is a need to ensure adequate oversight and high ethical standards of recruitment whichever approach is adopted. We believe that the model we have proposed offers significant additional protection to current by requiring the agreement of a specific policy on consent/assent practice with the ethics committee and the implementation of a reporting procedure. We also believe that a model requiring the assessment of the competence of individual children offers additional protections to one which rests upon a somewhat arbitrary age distinction. The requirement to make a judgement about competence in the case of young people is no different in principle from the requirement to pay attention to the competence of adult participants, even though the challenges are greater. If it is agreed that competence is the morally significant distinction in relation to consent, it ought to be this distinction that drives policy and practice in research ethics.

5. If taken in the wrong spirit, the proposed approach has the potential to erode rather than enhance children’s rights.

One possible and potentially worrying scenario would be where a competent child lived in a setting where he or she was not generally allowed to make decisions for which he or she was competent. The weight placed on contextual factors would seem to suggest that such a child should not be required to consent. Conversely, in a context in which children were forced by circumstance to make adult decisions which are beyond their competence and maturity e.g. where young children have to look after their siblings, our proposal might impose further inappropriate difficult decisions that they are not prepared to make. In addition to the above, children might be wrongly judged to be competent when they are not, and they may inadvertently choose to take on risks that that they do not comprehend.

Our position is that children who are competent to do so should provide their own consent (or refusal) to participate in research – the required level of competence being relative to a specific decision. The key moral distinction is between those children who are competent and those who are not. Our reference to the role of evidence about the kinds of decisions the child would normally be making outside the research context is intended to suggest a pragmatic approach to judgements about when and under what circumstances a child ought or ought not to be asked to consent in their own right. The assessment of competence is inevitably difficult in many cases and requires careful experienced judgement. Nonetheless, where it is judged that the decisions a particular child is being required to make (or barred from making) are not in fact indicative of the child’s level of competence, this should be taken into consideration.

## Summary

The concept of assent in the current guidelines is confusing. There is an urgent need for the development of both clearer guidelines which can be adapted for all types or paediatric research wherever they are done and an evidence-base about good assent/consent practice. In recognition of the benefits of pediatric research, research ethics has in recent years shifted from a position of excluding children from research to one of cautious advocacy – acknowledging the importance of pediatric research, but requiring this to be accompanied by careful consideration of the scientific and socio-economic context, evaluation of risks and benefits, and protection of participants. In this paper, we have argued that decision-making regarding the recruitment of children in research should be guided by two *prima facie* principles. Children who are competent (where competence is understood as relative to a specific decision in a specific context and judged against the background of locally accepted decision-making practices and expectations) should consent for themselves. A key challenge of this approach will inevitably be how to address different views about and practices regarding childhood competence in different settings and how to prevent abuse. Secondly, in the case of children lacking decision-making capacity (similarly contextualized), both the child’s assent and parental (or locally relevant alternative) consent should be obtained.

Finally, we have argued that there is a pressing need for further empirical work and ethical analysis in this important area. The issues we have identified and discussed in this paper were motivated by our own experience and discussion with others involved in research. As mentioned in the introduction of this paper, there is a large amount of important diversity between and within low-income settings. Further work is required to explore the ways in which issues in consent and assent arise differently in different places.

## Endnotes

^a^For the purposes of this paper we adopt the World Bank definition of low-income settings http://data.worldbank.org/about/country-classifications (accessed 25/07/13). Whilst recognizing that low-income settings exist in low-, middle- and high-income countries, our primary focus here is on low-income countries. For this reason, we adopted a practical set of criteria for low-income settings i.e. the income in the community is below the country average, there is a general lack of basic healthcare and educational facilities, and there is a high prevalence of diseases of poverty.

^b^This definition is based on that in the UK Mental Capacity Act 2005 and the UK Medical Research Council Ethics Guide: Medical Research Involving Children 2004.

## Competing interests

The authors declare that they have no competing interests.

## Authors’ contributions

PYC and MP contributed equally to the preparation of this manuscript. Both authors read and approved the final manuscript.

## Pre-publication history

The pre-publication history for this paper can be accessed here:

http://www.biomedcentral.com/1472-6939/15/22/prepub

## Declaration

This paper was presented in part at the American Society of Hygiene and Tropical Medicine Annual meeting in Washington D.C. in November 2013.
